# Editorial: Platelets in Disease: From Biomarkers Discovery to Therapeutic Targets

**DOI:** 10.3389/fphar.2022.860652

**Published:** 2022-02-14

**Authors:** Fan Zhao, Si Zhang, Shenghui Zhang

**Affiliations:** ^1^ Laboratory Animal Center, The First Affiliated Hospital of Wenzhou Medical University, Wenzhou, China; ^2^ Department of Biochemistry and Molecular Biology, NHC Key Laboratory of Glycoconjugates Research, School of Basic Medical Sciences, Fudan University, Shanghai, China; ^3^ Department of Hematology, Wenzhou Key Laboratory of Hematology, The First Affiliated Hospital of Wenzhou Medical University, Wenzhou, China

**Keywords:** platelets, biomarkers, therapeutic targets, hematologic toxicity, platelet hyperactivity

Platelets are the smallest non-nucleated cells in the blood circulation, and their specific morphological structure and biochemical composition make them play a key role in physiological hemostasis and pathological thrombosis (Zhao et al., 2021). Platelets are also widely recognized as novel biomarkers and therapeutic targets for related diseases such as inflammation, immunity and tumors.

Hematologic toxicity is a common adverse event associated with chimeric antigen receptor (CAR) T-cell therapy (Rejeski et al., 2021). Liu et al. showed that high incidence of platelet transfusion refractoriness (PTR) occurred in CAR-T therapy for relapsed/refractory acute lymphoblastic leukemia (R/R ALL), and the levels of IL-6 and IFN-*γ* in cytokine release syndrome (CRS) were positively correlated with PTR. PTR is a high-risk factor for hemorrhagic death. Endothelial activation drives the mechanism of CAR-T cell-mediated toxicity, suggesting that common laboratory parameters prior to lymphocyte depletion correlate with CAR-T-related toxicity (Greenbau et al., 2021). Therefore, an in-depth understanding of the mechanism of PTR can significantly counteract the risk of hemorrhagic death in CAR-T cell therapy, thereby improving the transfusion efficiency and disease treatment outcomes.

Zhang et al. showed that platelet hyperactivity in Crohn’s disease (CD) was associated with activation of the NLRP3 inflammasome. Firstly, CD patients had higher platelet counts, C-reactive protein (CRP) levels, erythrocyte sedimentation rate (ESR), and plateletcrit (PCT) levels than healthy volunteers. Secondly, the platelet sensor-NLRP3 and adaptor-ASC were found to have elevated mRNA and protein levels in patients with CD, which promoted the production of interleukin-1β. Thirdly, ROS levels elevated in patients with CD were associated with the platelet NLRP3 inflammasome. Although only a few cases were collected in this study, it provided a basis for further research on the mechanism of platelet hyperactivity in patients with CD. The significant increase in platelet activation in patients with sepsis may also be due to NLRP3 activation in platelets (Borges-Rodriguez et al., 2021). Studies on the pathogenesis and pathophysiology of primary immune thrombocytopenia (ITP) have shown that platelet NLRP3 inflammasome activation, due to reduced intracellular antioxidant capacity, plays a key role in ITP and may have potential diagnostic or therapeutic significance (Wang et al., 2021).

With in-depth research on platelets, targeted platelet therapy is expected to become an important strategy for the treatment of related diseases. Immune responses to SARS-CoV-2 peptides in whole blood assays are associated with COVID-19. And FcγRII and GPIIb/IIIa receptors also play platelet procoagulant roles in vaccine-induced thrombotic thrombocytopenia (VITT) (Petrone et al., 2021; Pitkänen et al., 2021). A model for the multiple interactions between SARS-CoV-2 and platelets was proposed by Cox. In this model, the SARS-CoV-2 spike protein binds to ACE2 expressed in platelets, while anti-SARS-CoV-2 antibodies can bind to platelet receptors such as FcγRIIa. After activating the combination of all these receptors, platelets are activated and eventually formed thrombi. The establishment of this model provides a deeper understanding of the multi-system disease caused by the interaction of COVID-19 and platelets, as well as a reasonable explanation to the adverse reactions generated in vaccines, which also provides and a research basis for targeted platelet therapy for severe infectious complications.

Matrine can reduce cardiac fibrosis, enhance cardiomyocyte viability, and attenuate inflammatory responses (Liu et al., 2021; Zhang et al., 2021). Zhang et al. used matrine as a new drug for the treatment of thrombosis and cardiovascular disease. Experiments have indicated that matrine may inhibit the production of reactive oxygen species through direct and indirect effects, thereby inhibiting platelet function, hemostasis, and arterial and venous thrombosis (Zhang et al.). Although matrine can be used as an antithrombotic agent, its potential bleeding risk remains unresolved.

Platelet function tests provide an important basis for hemostasis, thrombosis, and platelet dysfunction. Zhang et al. summarized routine platelet function tests in the market and introduced recent advances in novel microfluidic simulations of vascular anatomy. New microfluidic technologies inspired by platelet mechanobiology not only enable rapid analysis of blood disorders, but also drug testing and treatment evaluation in patients.

Hermansky-Pudlak syndrome (HPS) is characterized by oculocutaneous albinism (OCA) and bleeding diathesis due to a deficiency in melanosomes and platelet delta (*δ*)-granule secretion. Doris Boeckelmann et al. reported three patients with different HPS subtypes who suffered from bleeding diathesis with impaired platelet function and severely reduced platelet CD63 expression; however, only the patient with HPS-7 showed an apparent OCA. Panel sequencing identified several novel genetic variants in HPS3, HPS5, and HPS-7. The lack of apparent OCA features can result in delayed diagnosis of HPS. Even if the symptoms of OCA are evident, the diagnosis of HPS can be delayed if bleeding diathesis is not considered part of the disease. In the past, some infrequent subtypes of HPS were underdiagnosed. NGS improves the identification of HPS types even with a mild phenotype.

In conclusion, platelets have attracted increasing attention as new biomarkers for diagnostic and therapeutic strategies in immune, vascular and neoplastic diseases. The identification and quantification of platelet-related diseases is expected to become an important strategy for disease diagnosis in clinical applications, and the study of molecular mechanisms provides a more effective basis for the treatment of platelet-related diseases.
